# Amyloid-beta immunotherapy: the hope for Alzheimer disease?

**Published:** 2016-12-30

**Authors:** Alvaro Barrera-Ocampo, Francisco Lopera

**Affiliations:** 1 Departamento de Ciencias Farmacéuticas , Grupo de Investigación Natura, Facultad de Ciencias Naturales , Universidad Icesi , Cali, Colombia.; 2 Grupo de Neurociencias de Antioquia , Escuela de Medicina, Universidad de Antioquia, Medellin, Colombia.

**Keywords:** Amyloid beta-Peptides, antibodies, cognitive dysfunction, Alzheimer disease, adult, Immunotherapy, Vaccination, Immunization

## Abstract

Alzheimer disease (AD) is the most prevalent form of dementia of adult-onset, characterized by progressive impairment in cognition and memory. There is no cure for the disease and the current treatments are only symptomatic. Drug discovery is an expensive and time-consuming process; in the last decade no new drugs have been found for AD despite the efforts of the scientific community and pharmaceutical companies. The Aβ immunotherapy is one of the most promising approaches to modify the course of AD. This therapeutic strategy uses synthetic peptides or monoclonal antibodies (mAb) to decrease the Aβ load in the brain and slow the progression of the disease. Therefore, this article will discuss the main aspects of AD neuropathogenesis, the classical pharmacologic treatment, as well as the active and passive immunization describing drug prototypes evaluated in different clinical trials.

## Resumen

La enfermedad de Alzheimer (EA) es la forma más frecuente de demencia de inicio en el adulto, caracterizada por un deterioro progresivo en la cognición y la memoria. No hay cura para la enfermedad y los tratamientos actuales son sólo sintomáticos. El descubrimiento de fármacos es un proceso costoso y que consume mucho tiempo; en la última década no se han encontrado nuevos fármacos para la EA a pesar de los esfuerzos de la comunidad científica y las compañías farmacéuticas. La inmunoterapia contra Aβ es uno de los enfoques más prometedores para modificar el curso de la EA. Esta estrategia terapéutica utiliza péptidos sintéticos o anticuerpos monoclonales (mAb) para disminuir la carga de Aβ en el cerebro y retardar la progresión de la enfermedad. Por lo tanto, este artículo discutirá los principales aspectos de la neuropatogénesis de la EA, el tratamiento farmacológico clásico, así como la inmunización activa y pasiva describiendo los prototipos de fármacos evaluados en diferentes ensayos clínicos.

## Introduction

Dementia is a syndrome characterized by the loss or decline of memory and other cognitive functions such as speech, language, reasoning, judgment and thinking. Alteration in these functions interferes with performing everyday activities. It is estimated that in 2015 more than 46.8 million people worldwide had dementia and this number is expected to double every 20 years to 131.5 million in 2050 ^1^. These numbers are probably underestimated because they do not include individuals at early stages of the illness and the cases that are misdiagnosed. For these reasons, dementia is likely to become one of the most important health issues in the world.

Alzheimer disease (AD) is the most prevalent neurodegenerative disease of adult-onset, characterized by progressive impairment in cognition and memory. AD is also the most common type of dementia accounting for 60% to 80% of the cases ^2^. The largest number of affected individuals can be found in regions like the USA, Western Europe and China, and also in developing regions like western Pacific and Latin America ^3^. There are many other causes of dementia including cerebrovascular disease, dementia with Lewy bodies (DLB), mixed dementia (AD and vascular dementia, AD and DLB, and the combination of the three), frontotemporal lobar degeneration and Parkinson disease among others ^2^. Some aspects of these diseases overlap each other making difficult to identify the exact cause, thus the accurate diagnosis is a complex task. To appropriately diagnose AD, other forms of dementia need to be ruled out. This includes metabolic, endocrine and nutritional disorders (e.g., thyroid disease, vitamin B12 deficiency, and heavy metal poisoning); chronic infections, brain tumors, subdural hematoma, depression and medication-induced dementia ^4^. The disease has an average time course of 7 to 10 years and although the duration is different in every person with AD, symptoms seem to develop over the same stages. It is hypothesized that changes in the brain begin 10 to 20 years before any clinical manifestation appears. It has been established that AD starts with the neuronal death in the entorhinal cortex, a region that is connected with the hippocampus, which plays a major role in learning and is involved in transforming shot-term memories to long-term memories. The atrophy of these brain areas explains the symptoms of forgetfulness observed at the early stages of the illness, but other cognitive alterations, such as changes in attention and the ability to solve problems are present as well. The progression of the dementia to a mild stage last from 2 to 5 years and is evidenced by memory loss, language dysfunction, visuospatial difficulty, loss of insight and changes in the personality, among others. At this point, the person and the family become aware of the disease and the clinical diagnosis is usually made. In the moderate stage the damage has spread to the regions of the cerebral cortex that control language, reasoning, sensory processing and conscious though. Symptoms of the disease become pronounced and the person has behavioural problems, therefore more supervision is necessary. In AD, the disruption in the neuronal communication can cause hallucinations, delusions, paranoia, anger outbursts and also impaired ability to carry out routine tasks (e.g., bathing, dressing, reading, writing and working with numbers). This stage of moderate AD usually last from 2 to 4 years. The severe stage of AD is characterized by a widespread atrophy of the cerebral cortex and the enlargement of the ventricles. The person becomes completely dependent on caregivers because it is incapable of recognize the family and friends. The individual is unable to swallow, control bladder or bowel function, walk and sleep.

The causes of the AD are still unknown, but the scientific community agrees that multiple factors are involved in the disease progression and that a simple cause is improbable. Several risk factors have been shown to be related with the development of AD. Advanced age is the greatest risk factor for AD and most of the patients are aged 65 or older ^5^. Other risk factors include family history, being an Apolipoprotein E-ε4 (APOE-ε4) allele carrier, mild cognitive impairment (MCI), cardiovascular disease risk factors (high cholesterol, type 2 diabetes, high blood pressure, smoking, obesity, etc), and traumatic brain injury 6-10. In addition to the factors mentioned before, there is evidence that environmental risk factors, such as air quality, toxic heavy metals and occupational-related exposures, may also contribute to the development of AD 11.

The identification of risk factors is a key step in the early diagnosis of AD. In the last decade several tools have been developed to monitor the onset and the progression of the disease. There is an increasingly number of biomarkers (specific biomolecules present in blood or cerebrospinal fluid or imaging techniques) that allow to identify cellular and brain changes years before the first clinical symptoms of dementia begin. The current disease biomarkers focus on measuring levels of A β 40, A β 42 and Tau protein in cerebrospinal fluid. Imaging studies (MRI or PET) usually complement the analysis of fluid biomarkers. The introduction of the radiolabeled Pittsburgh Compound B (PiB), which binds to A β plaques in the brain, has allowed to track the aggregation process using PET scans ^12^. Recently, PET Tau imaging has been developed, this technique has great promise as a biomarker and may be useful to estimate the disease stage ^13^ ^,^ ^14^.

There is no cure for the disease, the treatments available are only symptomatic and the efficacy decays as the neurodegeneration progresses. The Aβ immunotherapy is one of the most promising approaches to modify the course of AD, therefore this review will discuss the main aspects of the active and passive immunization describing drug prototypes evaluated in different clinical trials.

## Alzheimer disease: Pathogenesis and Immunotherapy

### Neuropathogenesis of Alzheimer Disease

The most important pathological hallmarks of AD are senile plaques and neurofibrillary tangles (NFTs). The first are extracellular aggregates of Aβ peptides and the latter are intracellular aggregates of hyperphosphorylated Tau protein, a microtubule associated protein ^15^. Whereas in the amyloid cascade hypothesis genetic, pathologic, and biochemical evidence implicate aggregation of A β as a critical early trigger in the chain of events that lead to tauopathy, neuronal dysfunction, and dementia ^16^, the degree of Tau deposition correlates with the cognitive decline in AD ^17^ ^,^ ^18^ questioning the role of Aβ deposition as the trigger for Tau pathogenesis. Initially, the amyloid hypothesis stated that the neuronal dysfunction and death was produced by the toxic effects of the total A β load. Recently it has been suggested that not only A β elimination, but also its production can be altered in AD patients. Moreover, new studies indicate that not only A β peptides (A β 40 and Aβ42) contribute to the neuronal dysfunction, but that the oligomeric forms of the protein (small aggregates of two to 12 peptides) are actually more deleterious to brain functions than the Aβ aggregates such as senile plaques ^19^ ^,^ ^20^. Aβ peptides can also grow into fibrils, which arrange themselves into β-pleated sheets to form insoluble fibers of amyloid plaques ^21^.

Post-mortem analyses of human brains reveal a characteristic progression of Aβ plaques and a regular pattern of appearance of NFT. The progression of Aβ plaques appearance is correlated functionally and anatomically with affected brain regions ^22^ ^,^ ^23^. Aβ aggregation affects first the layers II-V of the isocortex, followed by the entorhinal cortex, hippocampal formation, amygdala, insular and cingulated cortices; it spreads then to the subcortical nuclei including striatum, basal forebrain cholinergic nuclei, thalamus, hypothalamus, and white matter. While the NFTs arise first in the locus coeruleus, entorhinal cortex and limbic brain areas such as the subiculum of the hippocampal formation, the amygdala, thalamus, and claustrum, and then spread to interconnected neocortical regions ^18^ ^,^ ^24^. The incidence of plaques and tangles correlates positively in AD, but until now there is no anatomical relationship between lesions.

### Molecular mechanism of Amyloid Precursor Protein (APP) processing

**Figure 1:**
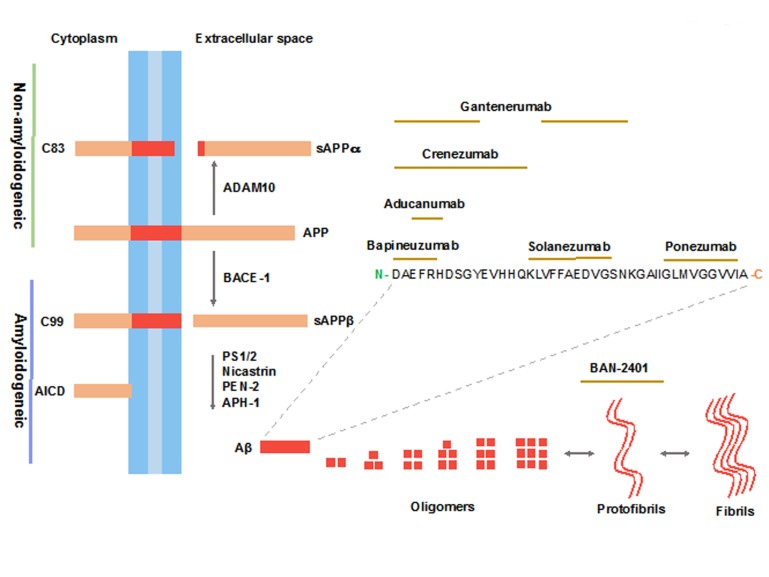
Processing of APP and Aβ mAb epitopes. In the Non-amyloidogenic pathway APP is first cleaved by α-secretase (ADAM-10) producing two fragments, sAPPα and C83, the late is cleaved by the γ-secretase complex generating the p3 and AICD peptides. The Amyloidogenic pathway involves the cleavage of APP by β-secretase (BACE1) producing the sAPPβ and C99 fragments; C99 is then processed by the γ-secretase complex producing Aβ and AICD peptides. The Figure shows the epitope region within the Aβ sequence for sequence-derived mAb.

The proteolytic pathway involved in the processing of APP has been well characterized using several in vitro and in vivo models ^25^ ^,^ ^26^. APP is produced in large amounts in neurons and metabolized very rapidly ^27^. After sorting in the endoplasmic reticulum (ER) and Golgi, APP is transported to the axon and synaptic terminals. The processing of APP takes place in the trans-Golgi network (TGN) and from there can be transported to the cell surface or to endosomal compartments. Both steps are mediated by clathrin-associated vesicles. Once on the cell surface, APP can be proteolyzed by α-secretases and the γ-secretase complex in a process that does not generate Aβ and which is known as the Non-amyloidogenic pathway (Fig. 1). The other possibility is that APP can be reinternalized in clathrin-coated pits in endosomal compartments containing β-secretases and the γ-secretase complex. The result of the interaction with these enzymes is the production of Aβ, which is then released to the extracellular space or is degraded in lysosomes. This process is known as the Amyloidogenic pathway (Fig. 1) (^28^-^30^). The α-secretase cleavage is mediated by members of the family of desintegrin and metalloproteinase domain proteins (ADAM), with ADAM-9, -10, -17 and -19 being the most likely candidates ^31^ ^,^ ^32^. The α-secretase cleavage site lies within the Aβ sequence and, thus, avoids Aβ formation ^33^. The α-secretase enzymatic activity generates two fragments. The N-terminal fragment is called secreted APP alpha (sAPPα) and the C-terminal fragment (CTF) is called CTF83 due to the amount of amino acid residues of this peptide (Fig. 1). The corresponding cleavage of CTF83 by the γ-secretase complex generates a small peptide known as p3 ^34^. The beta-site APP cleaving enzyme 1 (BACE1) is the most important β-secretase in the brain and is responsible for production of the sAPPβ and the CTF99 fragments (Fig. 1). The subsequent processing of CTF99 by the γ-secretase complex leads to the formation of Aβ and the amino-terminal APP intracellular domain (AICD) (Fig. 1) ^34^ ^,^ ^35^. A group of proteins constitutes the γ-secretase complex. Four proteins are required for this complex: PS1 or PS2, nicastrin, presenilin enhancer 2, and anterior pharynx defective 1. γ-secretase cleaves APP in its transmembrane region to create Aβ40/Aβ42 (Fig. 1) or p3 and AICD59/57, a second cut at the ε-cleavage site produces the AICD50 fragment ^36^ ^,^ ^37^.

### Aβ peptides and aggregates

The Aβ peptide may be considered the main product of the proteolytic processing of APP. The peptide is found in both, healthy and AD human brain at nanomolar concentrations or even lower. There are various isoforms of Aβ that differ by the number of amino acid residues at the C-terminal region of the peptide. The isoform Aβ40 has 40 residues and is the most abundant Aβ specie in the brain of AD patients ^38^. Aβ42 is also related to the disease because is less soluble than Aβ40 and form aggregates faster ^39^. The Aβ peptides have a specific type of β-sheet arrangement that favours the polymerization and aggregation, leading to the formation of oligomeric species that diffuse through the interstitial fluids. Aβ monomers tend to aggregate and polymerize, forming oligomers, protofibrils and fibrils (Fig. 1). Studies have shown that these assemblies arise from low molecular weight Aβ (monomers or dimers) ^40^ ^,^ ^41^. However, there is controversy about the toxicity of the different Aβ forms. Results indicate that soluble oligomeric assemblies can inhibit electrophysiological activity that may be important for the formation and storage of memory, therefore this step seems critical for the development of AD ^42^. Oligomers also bind to N-methyl-D-aspartate (NMDA) receptor subunits inhibiting synaptic plasticity and disturbing calcium homeostasis ^43^ ^,^ ^44^, which causes neuronal death. Thus, targeting Aβ forms can delay the aggregation process and mitigate the cognitive dysfunction that is the primary symptom of the disease. 

### Sporadic and Familial Alzheimer Disease

The two basic variants of AD are sporadic (SAD) and familial (FAD). The sporadic AD is characterized by absence of inheritance pattern and according to the age of onset can be classified as either early-onset (before 60 years of age) or late-onset (after 60 years of age). Familial AD is characterized by autosomal dominant heritability; accounts for probably less than 1% of the AD cases, and the disease tends to develop before 60 years of age (early-onset) ^45^ ^,^ ^46^. In FAD the cause of the disease is a genetic mutation in the genes coding for the amyloid precursor protein (APP), presenilin-1 (PS1) or presenilin-2 (PS2) ^47^. Currently, over 32 different missense mutations have been found in APP. Mutations in APP account for 10% to 15% of FAD and most of the cases have an age of onset 45 years. An important number of the mutations occur at the secretase cleavage site or in the transmembrane domain. Examples of this are the "Swedish" (K670N>M671L) and the "London" (V717I) mutations which are among the most studied APP mutations that lead to the increased production of Aβ and development of AD ^46^. Regarding PS1, more than 180 mutations have been identified and are responsible for around 80% of FAD cases. These mutations cause the most severe forms of AD; they have complete penetrance and an early onset of ~45 years ^47^ ^,^ ^48^. Mutations in PS1 seem to increase the ratio of Aβ42 to Aβ40 as a result of an increased Aβ42 and decreased Aβ40 production ^49^. So far more than 390 families carrying PS1 mutations have been identified. However, the worldwide largest group of individuals bearing a missense PS1 mutation is consisting of around 6,000 members of Colombian kindred carrying the E280A mutation ^50^ ^,^ ^51^. In contrast to the mutation in the PS1 gene, missense mutations in PS2 rarely cause early-onset FAD. The onset age among affected members of a family varies highly ^52^. Currently mutations have been identified in six families ^53^. One of them results in the substitution of a valine for a methionine at the residue 393 (V393M) located within the seventh transmembrane domain ^54^.

The understanding of AD pathogenesis has been greatly influenced by FAD cohorts. Nevertheless, the etiology of SAD is heterogeneous and many aspects are still unknown. The lack of clarity on the molecular basis of the disease and the interaction of multiple factors (e.g., genetic, epigenetic and environment) account for the complexity of this AD variant. The gap in the knowledge about the disease, the lack of accurate tools for the early diagnosis, the uncertainty to assess the disease stages and the stiffness of the classical drug discovery process may have contributed to the failure of the therapeutic strategies tried during the last two decades. Moreover, the compounds developed so far have focus on single pathways, most of them targeting the amyloid cascade. Emerging evidence demonstrates that other molecular changes (e.g., expression of genes, lipid metabolism, activity of Erk and other kinases) occur in parallel to the aggregation of Aβ and Tau even years before the onset of the first symptoms ^55^ ^-^ ^57^. The comprehension of these phenomena should be extended in order to succeed in the search for new therapies against AD. 

### Standard pharmacological treatment for AD 

During the last decades many efforts have been done to find the ultimate treatment for AD. The strategies vary from palliative alternatives to more sophisticated disease-modifying strategies such as gene- and immunotherapy. The palliative treatments include inhibitors of the enzyme acetylcholinesterase, which decrease the breakdown of the neurotransmitter acetylcholine. Rivastigmine, galantamine and donepezil are currently approved for use in mild to moderate AD (Table 1) ^58^ ^,^ ^59^. They seem to temporarily improve cognition, behavioural symptoms and routine tasks ^60^. However, clinical trials showed that one-third of the individuals had measurable benefits, one-third worsened the symptoms during the first 6 months, and 29% discontinue the treatment because adverse effects ^60^ ^-^ ^62^. Another drug approved for the palliative treatment of AD is memantine, an antagonist of the NMDA receptor, which reduces the neurotoxicity mediated by these; thus improving cognition, behaviour and activities of the daily living (Table 1) ^63^. It is used for moderate to severe AD, but despite the benefits observed, the high drop-out rates and the lack of effect on disease progression have limited the trials examining this drug ^58^ ^,^ ^59^ ^,^ ^64^. The limited effectiveness of the drugs mentioned before and the need for therapeutic agents capable of modify the course of the disease instilled the exploration of other fields of the biomedicine revolutionizing the classical pharmacologic approach taken so far. 

**Table 1:** Standard pharmacological treatment for Alzheimer disease.

Drug	Company of origen	Action	AD Patient status	Efficacy
Donezepil	Eisai	AChI	Mild to moderate	Improve cog., beh., DL
Rivastigmine	Novartis	AChI	Mild to moderate	Improve cog., beh., DL
Galantamine	Janssen	AChI	Mild to moderate	Improve cog., beh., DL
Memantine	Allergan	NMDAR antagonist	Moderate to severe	Improve cog.

AChI: Acetylcholinestarase inhibitor; NMDAR: N-Methyl-D-aspartate receptor;

Cog: Cognition; Beh: Behaviour: DL: Daily life activities

Source: http://www.clinicaltrials.gov

### Active vs Passive Immunotherapy

Based on the amyloid theory, which places A β as the first and main pathogenic factor of AD, the attempts to decrease the neurodegeneration cause by Aβ species try to program the immune system of the own patient to get rid of the Aβ peptides preventing the formation of amyloid plaques. This is called Active Aβ-immunotherapy and uses synthetic full-length, or a fragment or a fragment of the protein, to stimulate the production of antibodies by the B cells. The antibodies neutralize Aβ peptides and the complex is cleared out the brain. In 2002, the first active AD vaccine (AN1792) developed by ELAN in Ireland and Wyeth in USA went through a phase IIa clinical trial. The vaccine contained the full-length A β42 peptide and showed some beneficial effects including less cognitive decline. However, the trial was suspended due to the development of meningoencephalitis in ~6% of the individuals treated with the vaccine ^65^ ^-^ ^67^. One of the most plausible explanations of the development of this inflammatory process is that one the excipients used in the preparation produced the exposition of the Aβ C-terminus region, which seems to activate the T-helper type 2 response ^67^. For this reason, the new vaccines do not include this region of the peptide. Currently, several vaccines are being developed; these include CAD106 designed by Novartis in Switzerland, ACI-24 created by AC Immune in Switzerland and UB-311 made by United Neuroscience Ltd in Ireland. CAD106 contains the Aβ1-6 peptide coupled with a carrier with copies of the bacteriophage QB protein coat for the induction of the immune response (Table 2). The phase I trials showed no clinical cases of meningoencephalitis. However, during phase IIa trials one patient had intracerebral haemorrhage, whereas four individuals presented imaging abnormalities which were related with Aβ ^68^, ^69^. ACI-24 is made of the tetra-palmytoylated Aβ1-15 peptide which favours the β sheet folding (Table 2). This design is able to induce the production of conformation-specific antibodies and is formulated as liposome membranes to elicit the immune response ^70^. Phase I/IIa have been started and the preliminary results are to be published (https://clinicaltrials.gov; Identifier: NCT02738450). UB-311 consist of the Aβ1-14 peptide in combination with UBITh^®^ helper T-cell epitope, which specifically induces the activation of Th-1 cells (Table 2). The vaccine will be tested in a phase IIa study evaluating the safety and immunogenicity in patients with mild AD (https://clinicaltrials.gov; Identifier: NCT02551809). Although the active immunotherapy has demonstrated some benefits for AD patients and there are high expectations of the ongoing clinical trials, the safety and the difficulty to treat adverse effects still raises concern and constitute the main drawback of this therapeutic approach. 

**Table 2:** Active and Passive immunotherapeutic approaches for Alzheimer disease.

Active Immunotherapy						
Vaccine	Company of origen	Target	Formulation Adjuvant	Clinical trial phase	AD Patient status	Result
AN1792	ELAN/Wyeth	Aβ_42_	QS-21, polysorbte 80	IIa-finished	Mild to moderate	No improvement
CAD106	Novartis	Aβ_1-6_	Bacteriophage Qb protein coat	III	Prodromal	NR
ACI-24	AC Immune	tetra-palmytoylated Aβ_1-15_ (β conformation)	Liposomes	II	Adults with Down syndrome	NR
UB-311	United Neuroscience Ltd	Aβ_1-14_	CpG/Alum	II	Mild	NR
Passive Immunotherapy						
mAb	Company of origen	Antigen or Epitope /IgG	Binding species	Clinical trial phase	AD Patient status	Result
Crenezumab	AC Immune/Genentech	Pyroglutamate- Aβ_1-15_ (A)/hIgG4	Oligomers, fibrils and plaques	II	Mild	Decreased Aβ levels
Bapineuzumab	Janssen/Pfizer	NT Aβ_1-5_ (E)/hIgG1	Monomer, fibrils and amyloid plaques	III	Mild to moderate	Stabilized Aβ levels
Ponezumab	Janssen/Pfizer	CT Aβ_40_ (E)/hIgG2a	Aβ_40_(monomers, oligomers and fibrils	II	Mild to moderate	Decreased Aβ levels
Solanezumab	Eli Lilly	Aβ_16-24_ (E)/hIgG1	Monomers(oligomers and fibrils	III	Mild	Decreased Aβ levels
Gantenerumab	Roche	NT Aβ_1-10_ and central region Aβ_18-27_ (E)/human IgG1	Monomers, oligomers and fibrils	III	Prodromal to mild	Decreased Aβ levels
Aducanumab	Biogen	NT Aβ_3-6_ (E)/human IgG1	Oligomers and fibrils	Ib	Prodromal to mild	Decreased Aβ levels
BAN-2401	Biogen/Eisai/ BioArctic	Aβ_42_ AM protofibrils (A)/hIgG1	Protofibrils	I	Mild	NR

A: Antigen; E: Epitope; hIgG: Humanized IgG; NT: N-terminal region; CT: C-terminal region; AM: Arctic mutation; NR: Not reported

Source: http://www.clinicaltrials.gov

The Passive Immunotherapy overcomes the problems of the active immunization by using monoclonal antibodies (mAb), which act through three mechanisms that take place once the antibody has crossed the blood-brain barrier ^71^ ^,^ ^72^. The first is mediated by the interaction Aβ-mAb decreasing the formation of toxic aggregates. The second requires the binding between the Fc domain of the mAb and the Fc-γ receptors present on the microglia leading to the phagocytosis of the Aβ-mAb complex. The third mechanism involves the activation of the complement-depend cytotoxicity effect by the Aβ-mAb complex producing the lysis of the target cell. There is a fourth mechanism of action in which the mAb interact with Aβ in the peripheral blood and creates a concentration gradient that causes the efflux of Aβ from the brain. Some mAb against Aβ that are being tested in clinical trials include bapineuzumab, ponezumab, solanezumab, gantenerumab, aducanumab, crenezumab and BAN-2401. It is important to notice that most of these clinical trials use a randomized design which entails limitations such as the lack of heterogeneity among participants and the difficulty that possess the cognitive evaluation across individuals with different gender, age and educational level among others. Despite these faults, this methodology is the "gold standard" for treatment efficacy studies and it can be supplemented to overcome some of the limitations mentioned above ^73^.

Bapineuzumab was the first antibody to be tested in clinical trials after the termination of the AN1792 clinical study. It consist of a humanized IgG1 mAb that binds to the N-terminal region of Aβ (Table 2) ^74^. The analysis of the phase II clinical trials in mild to moderate AD patients revealed modest improvement related to the stabilization of Aβ burden. However, some individuals treated with the antibody suffered reversible edema which is considered an Amyloid-related Imaging Abnormality (ARIA-E) ^75^ ^,^ ^76^. This event and the lack of clear benefits during the phase III led to the finalization of clinical trials.

Ponezumab is a humanized IgG2a mAb against the C-terminal epitope of Aβ, which has a much stronger binding to Aβ40 than to other monomers, oligomers or fibrils (Table 2). It diminishes the amyloid burden through an outflow of Aβ from the hippocampus induced by the reduction of the peptide in plasma ^77^. Results of the clinical trials evidenced no significant improvement in cognitive impairment of patients with mild to moderate AD ^78^ ^,^ ^79^ and at the moment is being tested for the treatment of cerebral amyloid angiopathy (CAA) (https://clinicaltrials.gov; Identifier: NCT01821118). 

Solanezumab is the humanized version of the m266 IgG1 mAb that binds the central region of Aβ and has more affinity to monomers than to soluble and toxic species in patients with mild AD (Table 2) ^80^. At first, the results of the phase III clinical trials did not demonstrate significant improvements in individuals treated with the antibody ^81^. A complementary data analysis revealed less cognitive and functional deterioration in AD patients ^82^. The antibody has been well tolerated, but ARIA-E has been observed in 16 individuals enrolled in double-blind trials (EXPEDITION and EXPEDITION 2) and their ongoing open-label extension trial (EXPEDITION-EXT) ^83^. In addition, the magnitude of the benefits is at the same level of the inhibitors of acetylcholinesterase (https://clinicaltrials.gov; Identifier: NCT02760602 and NCT02008357). Disappointedly, the last 23 of November Eli Lilly announced that it would abandon the clinical trials on the drug because the results of the EXPEDITION3 study showed that solanezumab was not able to slow down cognitive decline in patients with AD compared with those who received placebo. One plausible explanation for the failure is that the antibody could be trapped in the blood and does not reach therapeutic concentrations in the brain ^84^. 

Gantenerumab was first fully human mAb designed to bind with subnanomolar affinity to a conformational epitope on Aβ fibrils. It encompasses both N-terminal and central amino acids of Aβ binding to monomers, oligomers and fibrils in individuals with prodromal to moderate AD (Table 2) ^85^. The antibody reduces the amyloid load and activates the microglia avoiding the plaque formation ^85^. During the phase I clinical trials the antibody was safe and well-tolerated; however, some patients treater with high dosages developed transient ARIA ^86^. The phase II studies indicated not efficacy in the enrolled cohort, but post-hoc analysis showed a slight benefit in patients with fast progression. At this moment phase III clinical trials are in course, these include a study to evaluate the effect of the antibody on safety, pharmacokinetics, cognition and functioning in individuals with prodromal AD (https://clinicaltrials.gov; Identifier: NCT01224106). A trial to test the efficacy and safety of gantenerumab in patients with mild AD (https://clinicaltrials.gov; Identifier: NCT02051608); and a phase II/III study to determine whether the antibody improves the cognitive outcome of participants with dominantly inherited AD (https://clinicaltrials.gov; Identifier: NCT01760005). 

Aducanumab is a human IgG1 mAb developed from a B-cell library created from healthy aged individuals ^87^. The antibody interacts with the Aβ N-terminal region binding to oligomers and fibrils of subjects with prodromal to mild AD (Table 2) ^87^ ^,^ ^88^. The phase Ib clinical trial showed improvement of cognitive decline, but caused ARIA in patients with high-dose treatment. The limitations of this study included small sample sizes, the use of sequential dose-escalation design and it was not powered by exploratory clinical endpoints. The trial proved safe and effective in amyloid clearance, but the positive effects of cognition were less clear ^87^. Based on interim data analysis and the promising results, it was decided to start two phase III studies set to evaluate the efficacy of aducanumab in slowing cognitive and functional impairment in participants with early AD. The trials will run until 2022, in 150 centers in North America, Europe, Australia, and Asia (https://clinicaltrials.gov; Identifier: NCT02477800 and NCT02484547). The expectative for results of these trials is great because they can truly put the amyloid hypothesis to the test. 

Crenezumab, also known as MABT, is a humanized antibody directed against the mid-region of Aβ that uses an IgG4 isotype to reduce the risk of microglial overactivation. It recognizes Aβ monomers, oligomers and fibrils, even though it has less affinity for the first (Table 2) ^70^ ^,^ ^89^. Currently, the "Alzheimer's Prevention Initiative" (API) is recruiting 300 Colombian individuals, 200 harbouring the E280A PS1 mutation and 100 non-carriers ^90^. The purpose of the study is to evaluate the safety and efficacy of the antibody in a preclinical phase of AD (https://clinicaltrials.gov; Identifier: NCT01998841).

BAN-2401 is a humanized mAb directed against APP bearing the E22G mutation in Aβ (Arctic mutation) ^91^. The antibody is able to recognize a specific conformation in Aβ protofibrils (Table 2) ^91^. Phase I clinical trial proved that the antibody was safe and no serious adverse events were observed ^92^. A phase II study is currently enrolling participants to determine the clinical efficacy of BAN-2401 on mild cognitive impairment and mild AD (https://clinicaltrials.gov; Identifier: NCT01767311).

## Conclusions

The immunotherapy works together with the human immune system to neutralize the aggregation process of Aβ species. Currently, it may be the best approach to modify the neurodegeneration and the cognitive decline present in AD. Nonetheless, more studies are necessary to find vaccines more specific that do not elicit the autoimmune response. Regarding passive immunization, the efficiency of mAb to cross the blood-brain barrier has to be improved, as well as the cross reactivity and the inflammatory alterations observed in some patients. It is also convenient to contemplate the use of non-immunogenic compounds such as DNA or RNA aptamers, which are small oligonucleotide fragments with strong affinity to diverse targets ranging from small molecules to cells and can overcome the problems observed with the immunization ^93^. 
